# Implications of Intra-Individual Variability in Motor Performance on Functional Mobility in Stroke Survivors

**DOI:** 10.3390/geriatrics10020051

**Published:** 2025-03-24

**Authors:** Neha Lodha, Prakruti Patel, Evangelos A. Christou, Anjali Tiwari, Manfred Diehl

**Affiliations:** 1 Movement Neuroscience and Rehabilitation Laboratory, Department of Health and Exercise Science, Colorado State University, Fort Collins, CO 80523-1582, USA; prakruti.patel@colostate.edu (P.P.); anjali.tiwari@colostate.edu (A.T.); 2 Department of Applied Physiology and Kinesiology, University of Florida, Gainesville, FL 32611-8205, USA; eachristou@ufl.edu; 3 Department of Human Development and Family Studies, Colorado State University, Fort Collins, CO 80523-1570, USA; manfred.diehl@colostate.edu

**Keywords:** intra-individual variability, stroke, functional performance, driving issues, motor control

## Abstract

**Background:** Motor impairments following stroke contribute to deficits in functional mobility. Traditionally, these impairments are quantified by mean-level motor performance. However, this mean-level approach neglects the well-established fact that motor performance becomes highly variable in aging and disease. Increased intra-individual variability (IIV) in behavior predicts functional decline in neurological disorders. Despite this, the impact of stroke on IIV in motor performance and its influence on functional mobility has not been investigated. This study aimed to (1) quantify the impact of stroke on IIV in motor performance, and (2) determine the contribution of IIV and mean motor performance to functional mobility. **Methods**: Twenty stroke survivors and 20 age-matched controls performed a goal-directed ankle movement task over 30 trials. We measured average accuracy (mean endpoint error) and IIV (within-person SD of endpoint error). Functional mobility was assessed with postural control (sway area during quiet standing) and braking response time in a driving simulator. **Results**: Stroke participants showed a higher mean (*p* = 0.04) and greater IIV (*p* = 0.016) in endpoint error than controls. Sway area did not differ between groups (*p* = 0.24), but stroke survivors had increased braking response time (*p* = 0.016). In stroke survivors, IIV significantly predicted sway area (R^2^ = 0.33, *p* = 0.008) and braking response time (R^2^ = 0.27, *p* = 0.02), and mean error did not account for any additional variance. **Conclusions:** Stroke reduces the trial-to-trial consistency of executing motor tasks with precision. IIV in motor performance predicts postural balance and braking response time and can potentially serve as an indicator of increased vulnerability and an important target for stroke rehabilitation.

## 1. Introduction

Motor impairments following stroke have a significant impact on functional mobility [1,2]. Typically, stroke-related motor impairments such as decreased strength, speed, and accuracy have been quantified by measuring mean-level performance across multiple trials [3,4]. The mean serves as a central tendency measure to provide a general estimate of an individual’s motor performance. However, this conventional approach to measuring mean-level performance neglects the well-established fact that motor performance becomes highly variable following neurological disorders, rendering mean-level data less meaningful [5,6,7]. Because of the diverse etiology and symptoms related to stroke, fluctuations in performance across trials are highly probable [5,8]. These trial-to-trial fluctuations in performance, reflecting intra-individual variability (IIV) in motor behavior (termed here inconsistency), warrant systematic investigation in the stroke population. In this context, we define inconsistency as heightened within-person instability across repeated attempts of a motor task.

Intra-individual variability is a key behavioral indicator of underlying alterations in neurobiological integrity, including diminished white matter volume, heightened neural noise, and impaired neurotransmission [9,10,11]. Seminal work on lifespan psychology has shown that IIV in behavioral performance is a classic feature of both progressive and acute neurological insults [12,13,14]. For example, IIV in processing speed is exacerbated following traumatic brain injury [12,13], Parkinson’s disease [14,15], and multiple sclerosis [16,17]. Although intra-individual variability in cognitive performance is well studied across several age-related neurological conditions [15,18,19], the influence of stroke on trial-to-trial variability in motor performance has not been well established. We posit that if diminished neural integrity contributes to behavioral fluctuations, then IIV in performance may be a generalized feature across multiple domains, including motor function.

Previous studies have shown that individuals with stroke exhibit higher within-trial variability while executing upper-limb and lower-limb movements [20,21,22]. Given that variability within a single trial and variability across multiple trials can be theoretically unrelated to each other [7], it is unclear to what extent stroke impacts trial-to-trial motor variability [23]. A few studies have examined trial-to-trial variability in reaching movements post-stroke, although the focus was on task-dependent modulation of variability without clear implications on functional performance [24,25]. Characterizing stroke-related fluctuations in motor performance over successive trials and its impact on functional capacity is critical for identifying mechanistic targets for rehabilitation interventions following stroke.

Increased IIV in cognitive performance has emerged as a sensitive predictor of functional decline and adverse health outcomes, above and beyond mean-level measures in healthy older adults and at early stages of Alzheimer’s disease [26,27]. Recent evidence indicates that stroke survivors with heightened IIV in cognitive performance self-report greater difficulty in performing activities of daily living [8]. Despite subjective evidence suggesting that increased IIV following stroke could negatively impact everyday function, the potential influence of heightened IIV on functional capacity post-stroke remains largely unexplored. Increased IIV is indicative of executive dysfunction and impaired attentional control [28,29], both of which are risk factors for reduced mobility in older adults [30,31]. Therefore, we propose that IIV may influence functional mobility in individuals with stroke. In this study, we focus on balance and driving as key forms of functional mobility in older adults [32]. Recovery of adequate balance is linked with greater independence, enhanced performance in daily activities, and higher quality of life in stroke survivors [33]. Similarly, the ability to drive safely significantly impacts functional autonomy, work, social engagement, and community integration after stroke [34].

The goal of the current study was twofold: (1) to quantify the impact of stroke on intra-individual variability in motor performance; and (2) to determine the contribution of intra-individual variability and mean motor performance to functional mobility as assessed by postural sway and simulator driving. We examined the mean and within-person standard deviation (SD) of motor accuracy during goal-directed ankle dorsiflexion movements. Hierarchical multiple linear regression was performed to examine whether the mean and/or IIV of the endpoint error predicts sway area and braking response time in individuals with stroke. We hypothesized that IIV in motor performance will be increased in individuals with stroke compared with healthy older adults. Based on previous research in aging [35], Parkinson’s disease [15], and Alzheimer’s disease [36], we hypothesized that IIV in motor performance would have greater predictive relevance for functional mobility in stroke survivors than mean-level motor performance.

## 2. Materials and Methods

### 2.1. Participants

Twenty individuals with chronic stroke and twenty healthy control participants volunteered to participate in the study. Participants were recruited from the north-central Florida region and surrounding areas, regardless of their gender or ethnicity between July 2014 and August 2016. Table 1 shows the clinical characteristics of all the participants. Control participants were healthy older adults without any neurological impairments. Inclusion criteria for the stroke participants were (1) diagnosis of a single, unilateral cerebrovascular accident at least 9 months prior to participation in the study; (2) ability to perform plantarflexion and dorsiflexion of the paretic ankle on command; (3) a minimum of 10 degrees of active ankle movement (combined dorsiflexion and plantar flexion); (4) current or previous drivers, (5) be able to drive in the driving simulator, and (5) ability to stand without supervision for 30 s. Exclusion criteria for all participants included self-reported presence of any other neurological or musculoskeletal disorder, uncorrected visual and hearing impairments, visual neglect, aphasia, and pain in the lower extremity or elsewhere that could interfere with standing or ankle movements. Considering the inclusion/exclusion criteria necessary for performing the functional mobility tasks, predominantly individuals with high functional capacity were eligible for participation. We recruit individuals 9 months post-stroke because typically outpatient rehabilitation is complete by this stage and most individuals are no longer undergoing active physical intervention that may affect IIV in a systematic way. University of Florida’s Institutional Review Board approved all experimental procedures. All individuals read and provided written informed consent prior to participation.

### 2.2. Experimental Protocol

Each experimental testing session lasted ~90 min and was conducted at University of Florida. At the beginning of each task, we explained the experimental procedures to the participants. During the experimental session, we conducted the following: (1) clinical assessments that included Fugl–Meyer Motor Assessment for lower extremity, the Montreal Cognitive Assessment, range of motion, and Driving Habits Questionnaire; (2) goal-directed movements with ankle dorsiflexion; and (3) functional mobility tasks that included postural balance and driving in a simulator. The assessments were conducted by a research assistant while data analysis was conducted by authors to prevent assessment bias.

### 2.3. Experimental Procedures

Clinical assessments: Fugl–Meyer Motor Assessment (FMA): we assessed the severity of leg and foot motor impairments of the stroke participants, using the lower extremity subsection of the FMA [37]. The FMA ranged between 0 and 34 such that lower scores indicated more severe motor impairments. Montreal Cognitive Assessment (MoCA): we assessed global cognitive function across multiple domains using the MoCA [38]. The MoCA ranged between 0 and 30 such that a score of 26 or less is indicative of cognitive impairment. Range of motion (ROM) assessment: we measured ankle range of motion using a goniometer to ensure that participants had adequate ROM to perform the goal-directed task. The Driving Habits Questionnaire (DHQ) determined the self-reported behavior related to driving exposure, driving space, driving avoidance, and citations [39]. The DHQ score ranged between 0 and 15 such that a higher score indicated greater involvement in on-road driving behavior.

### 2.4. Goal-Directed Movements

The protocol for rapid goal-directed ankle movements was adapted from previously published work [40,41,42,43,44,45,46] from our group.

**Figure 1:**
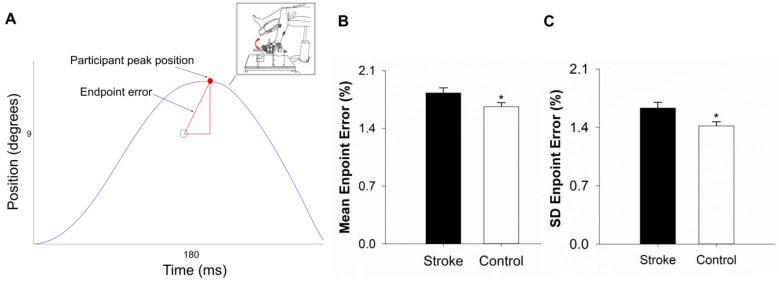
Goal-directed movement: (**A**). Inset: Experimental setup for goal-directed ankle dorsiflexion movement. Participants were required to perform 9° of dorsiflexion (position target) with 180 ms (time target). A representative participant’s performance on a single trial relative to the target. (**A**). The participants in the stroke group showed an increased mean of endpoint error (**B**) and increased intra-individual variability (within-person standard deviation; SD) of endpoint error (**C**) compared with the participants in the control group. * *p* < 0.05.

Apparatus: The participants sat upright facing a 32-inch monitor (Sync Master 320MP-2, Samsung Electronics America (Ridgefield Park, NJ, USA), Resolution: 1920 × 1080, Refresh rate: 60p Hz). The monitor was located 1.25 m away at eye level. Before beginning the task, all participants confirmed that they could see the screen clearly. The hip joint was flexed to 90° with 10° abduction, the knee was flexed to 90°, and the ankle was plantarflexed to 10°. The foot was placed on a customized device that only allowed for dorsiflexion and plantarflexion. To ensure simultaneous movement of both the foot and the device, the participant’s foot was secured by strapping the metatarsals to the device (Figure 1A-inset).

Protocol: Participants performed 3–5 practice trials of goal-directed movement to ensure they understood the task and could complete rapid ballistic ankle dorsiflexion. Individuals with stroke performed the task with their paretic leg and controls performed this task with their non-dominant leg. After the practice trials, participants completed 30 sequential goal-directed movements. The target was an ankle dorsiflexion movement of 9° in 180 ms. This target was chosen to ensure the task’s rapid nature required participants to pre-plan their movements, relevance to ankle control involved in driving, and accommodating age-related decline in ankle range of motion for task feasibility [47,48]. This was not a reaction time task, but rather a controlled fast contraction task, such that the timer began only after the participant initiated the movement. No other instructions were provided to allow participants to plan their own movements.

Movement Task: This task had three phases GET READY; MOVE; and FEEDBACK. In the GET READY phase, a red target was presented on the monitor for 2 s. This prompted the participants to prepare for the next phase. In the MOVE phase, the red target changed colors to green. The green target was shown on the monitor for 3 s, prompting participants to execute the goal-directed movement. Participants were instructed to begin the movement within the 3 s of appearance of the green target but were not required to begin the movement as soon as the green target appeared, because this task was not a reaction time task. The movement time recording began as soon as the participant initiated the movement. We did not provide online feedback on the movement to eliminate adjustments while performing the task. The FEEDBACK phase started at the end of the MOVE phase and lasted for 5 s. This phase provided the participants with visual feedback on their performance (movement trajectory) relative to the target position and time. The feedback allowed participants to see how they performed in the current trial (Figure 1A) and plan the movement for subsequent trials.

Data acquisition: The ankle position was measured using a low-friction potentiometer (SP22G-5K, Mouser Electronics, Mansfield, TX, USA) placed in line with the fibular malleolus. The ankle position data were sampled at 1000 Hz using a NI-DAQ card (model USB6210, National Instruments, Austin, TX, USA) and stored on a personal computer for offline data analyses.

Endpoint Error: To calculate the endpoint error, we first quantified the position and time errors. Position error was computed as the absolute vertical deviation from the targeted position to the peak position displacement. The time error was quantified as the absolute horizontal deviation from the targeted time to the peak time displacement. Position and time errors were normalized to have similar units (%). The position error was normalized to the target position of 9° and the time error was normalized to the target movement time of 180 ms. The endpoint error was quantified as the hypotenuse between the position error and time error distance (Equation (1); Figure 1A).

(1) $$Endpoint\;error\;\left(\%\right)=\sqrt{{\left(time\;error\right)}^{2}+{\left(position\;error\right)}^{2}}$$

Mean endpoint error: To quantify the mean endpoint error for each participant we averaged the endpoint errors across the 30 trials. This variable measured each participant’s mean-level performance on the goal-directed task.

Intra-individual variability in endpoint error: To quantify the intra-individual variability (IIV) of motor performance, we computed for each participant the within-person standard deviation (SD) of the endpoint errors across the 30 trials on the goal-directed task. The within-person SD measured each participant’s IIV in motor performance during the goal-directed task.

### 2.5. Postural Balance

**Figure 2:**
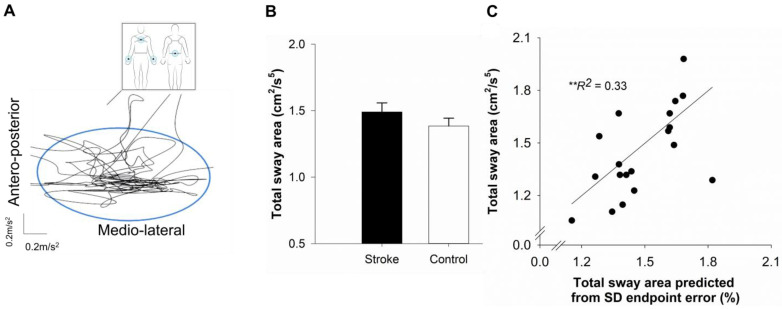
Postural Balance: (**A**). Inset: Participants wore six wearable sensors on both wrists, both ankles, sternum, and lumbar region and stood in a quiet stance for 30 s. We measured the sway area during the 30 s period. The black line shows the sway trajectory of a participant during a single trial (**A**). The blue line represents the sway area. The sway area did not differ significantly between the stroke and the control group (**B**). Hierarchical regression analysis showed that the intra-individual variability (within-person standard deviation; SD) of endpoint error predicted sway area (**C**). ** *p* < 0.01.

Task: Participants performed a quiet standing task while looking straight ahead at an empty corridor for 30 s. Six inertial sensors (APDM Inc., v1, Portland, OR, USA) were placed on both wrists, both ankles, the lumbar spine, and the sternum. These accelerometer-based inertial sensors worn by the participants measured the static balance. Three trials were performed with a 90 s rest period between trials. Figure 2A shows the schematic representation of the participant’s position and time series of the sway trajectory during the quiet standing task.

Sway area: We measured the static postural balance with the sway area. The APDM Mobility Lab program computed the sway area using a validated custom-developed algorithm [49,50]. The sway area averaged across three trials was used as the outcome variable for analyses.

### 2.6. Simulated Driving

**Figure 3:**
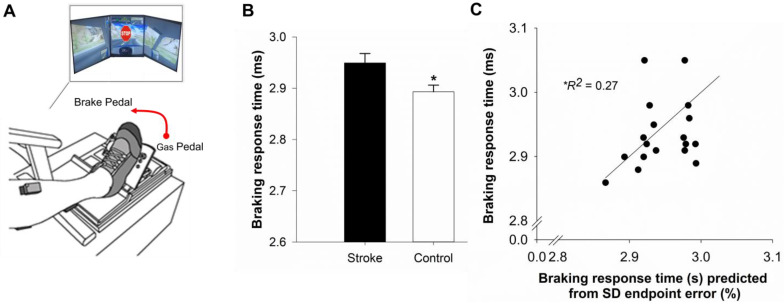
Driving in a simulated environment: (**A**). Inset: Driving environment displayed to the participants. Participants drove in a compact car and in response to a STOP stimulus presented at random intervals during the drive, they applied brake as fast as possible by moving the foot from the gas to the brake pedal. Gas and brake pedal positioning in the driving simulator (**A**). The braking response time was longer in the stroke group than in the control group (**B**). Hierarchical regression analysis showed that the intra-individual variability (within-person standard deviation; SD) of endpoint error predicted braking response time in the stroke group (**C**). All the variables were log-transformed prior to analyses and represented in the figure. * *p* < 0.05.

Experimental setup: Participants sat in an upright position in a professional driving simulator seat (AplusB software, Myrtle Beach, CS, USA) with a steering wheel, a gas pedal, and a brake pedal. The driving environment was presented on three 24-inch computer monitors located side by side at eye level. The driving scenario involved navigating a compact car along a winding road with oncoming traffic, in clear and sunny weather. The simulated driving task was performed with the paretic leg in the stroke and the non-dominant leg in the control group. Figure 3A shows the schematic representation of the participant’s position and simulated driving environment.

Simulated driving task: Participants were instructed to drive at 30 km/h. At random intervals during the driving course, a STOP stimulus would randomly appear. Participants were asked to respond to the STOP stimulus as quickly as possible by releasing the gas pedal and pressing on the brake pedal. To familiarize the participants with the simulated driving task, participants completed two practice trials. Following practice, each participant performed a 3 min simulated driving task during which 10 STOP stimuli were presented at random time intervals.

Braking Response Time: Braking response time was measured as the time between the presentation of the STOP stimulus and the application of the brake pedal. Application of the brake pedal was recorded when a 10% change in brake pedal position from the neutral position occurred. The braking response time was averaged across the 10 trials to obtain the mean braking response time.

## 3. Statistical Analysis

The Shapiro–Wilk test was used to assess the normality of the data distributions from the goal-directed task, the postural balance, and the simulator driving task for both groups. Because the variables were not normally distributed in either group, we log-transformed all variables and performed statistical analyses on the log-transformed values. We performed independent *t*-tests to compare the stroke and control group on mean and within-person SD of the endpoint error in the goal-directed task, sway area, and braking response time. Cohen’s *d* reports the effect size of the mean group differences. To examine associations among the mean, within-person SD of motor performance, sway area, and braking response time, Pearson’s bivariate correlations were computed. To determine whether the IIV of the endpoint error was an independent predictor of sway area and braking response time, we performed two separate multiple hierarchical regression analyses with sway area and braking response time as the criterion variables. In line with the hypothesis, the within-person SD of the endpoint error served as the predictor variable at step 1 and the mean endpoint error was added as a predictor variable at step 2 in both regression analyses. All statistical analyses were conducted with the alpha level set at 0.05. All statistical analyses were performed using SPSS 25.0 (IBM, Armonk, NY, USA).

## 4. Results

### 4.1. Clinical Characteristics

The participants in the stroke and control groups were similar in age (*t*_38_ = 0.10; *p* = 0.91; Table 1) and the distribution by sex was about equal. In the stroke group, the FMA lower extremity score (mean 26.39, SD 6.33) suggested a relatively moderate-to-mild level of motor impairment. The stroke group showed reduced scores on the MoCA (*t*_38_ = 4.34; *p* < 0.001; *d* = 1.39) and lower scores on the driving habits questionnaire (*t*_38_ = 14.61; *p* < 0.001; *d* = 4.62) compared with the participants in the control group.

### 4.2. Performance on Goal-Directed Task

Table 2 shows the raw values and Figure 1B,C show log-transformed values for the mean and the within-person SD of the endpoint error. The participants in the stroke group showed a higher mean on the endpoint error variable relative to the participants in the control group (*t*_38_ = 2.10; *p* = 0.04, *d* = 0.64; Figure 1B). The participants in the stroke group also showed a greater within-person SD on the endpoint error variable compared with the participants in the control group (*t*_38_ = 2.52; *p* = 0.01, *d* = 0.82; Figure 1C). A secondary co-variate analysis revealed that age and sex had no significant impact on the mean and within-person SD of the endpoint error (*p* = 0.37–0.97).

### 4.3. Performance on Functional Mobility Tasks

Table 2 shows the raw values and Figure 2B and Figure 3B shows log-transformed values for the sway area and braking response time. (4.3.*a*) *Postural balance:* although the sway area seemed to be increased within the stroke group, statistical analysis did not reveal significant differences between the two groups (*t*_38_ = 1.17; *p* = 0.24, *d* = 0.37; Figure 2B). (4.3.*b*) *Simulator driving*: the braking response time was significantly longer for participants in the stroke group compared to the participants in the control group (*t*_37_ = 2.52; *p* = 0.01, *d* = 0.72; Figure 3B).

### 4.4. Association Between the Functional Mobility Outcomes, Mean Endpoint Error, and IIV of Endpoint Error

In the stroke group, the sway area was significantly positively correlated to the within-person SD of the endpoint error (*r* = 0.57, *p* = 0.008), whereas the correlation with the mean endpoint error was not statistically significant (*r* = 0.31, *p* = 0.17). In the stroke group, the braking response time was significantly positively correlated with the mean (*r* = 0.48, *p* = 0.03) and within-person SD (*r* = 0.51, *p* = 0.02) of the endpoint error.

### 4.5. Predicting Sway Area Using Hierarchical Regression

To determine whether the IIV of the endpoint error and the mean of the endpoint error predicted the sway area, we conducted a multiple hierarchical regression. The IIV of the endpoint error was entered at step 1 and the mean endpoint error was entered at step 2 (see Table 3). The collinearity statistics were within the accepted limits (variance inflation factor (VIF) = 2.01), suggesting that the assumptions of multicollinearity between independent variables were met. Based on step 1, the IIV of the endpoint error accounted for 33% of the variance in the sway area (*R*^2^ = 0.33, *F*_1,19_ = 8.75, *p* = 0.008; Figure 2C). Based on Cohen (1988), R^2^ of 33% qualifies as a large effect size (R^2^ > 26%) [51]. The addition of the mean endpoint error in step 2 of the regression model did not significantly improve the model fit (Δ*R*^2^ = 0.017, Δ*F*_1,17_ = 0.43, *p* = 0.518), resulting in a total of 34.4% of the variance in the sway area accounted for by both predictors (*R*^2^ = 0.344, *F*_2,19_ = 4.45, *p* = 0.028).

### 4.6. Predicting Braking Response Time Using Hierarchical Regression

To determine whether the IIV of the endpoint error and the mean of the endpoint error predicted the braking response time, we conducted a second multiple hierarchical regression. The IIV of the endpoint error was entered at step 1 and the mean endpoint error was entered at step 2 (see Table 4). The collinearity statistics were within the accepted limits (VIF = 1.86), suggesting that the assumptions of multicollinearity between mean and within-person SD were met. At step 1, the IIV of the endpoint error accounted for 27% of the variance in the braking response time (*R*^2^ = 0.27, *F*_1,18_ = 6.13, *p* = 0.02; Figure 3C). R^2^ of 27% qualifies as a large effect size based on the guidelines provided by Cohen [51]. The addition of the mean endpoint error at step 2 did not significantly improve the model fit (Δ*R*^2^ = 0.031, Δ*F* _1,16_ = 0.71*, p* = 0.41), resulting in a total of 29.6% of the variance in braking response time being accounted for by both predictors (*R*^2^ = 0.296, *F* _2,18_ = 3.36, *p* = 0.06).

## 5. Discussion

The current study aimed to examine the impact of stroke on the intra-individual variability in motor performance, and its influence on functional mobility. The individuals with stroke showed a significantly greater within-person SD of the endpoint error during a goal-directed ankle dorsiflexion task. Thus, stroke influences intra-individual variability in motor performance by impairing the trial-to-trial consistency of executing movements with precision. Importantly, increased IIV in motor performance was a significant predictor of postural sway and braking response time in stroke survivors, whereas mean motor performance did not provide any additional predictive relevance. Overall, our findings provide novel evidence that stroke impairs trial-to-trial consistency of motor performance. Importantly, increased IIV in motor performance independently predicts postural balance and braking time following stroke. Further, the mean-level performance was not a significant predictor of functional mobility above and beyond IIV in motor performance. These findings highlight the importance of conducting repeated measurements to capture IIV in motor behavior and its impact on functional mobility following stroke [52].

### 5.1. Stroke Amplifies Intra-Individual Variability in Motor Performance

Everyday functional activities involve rapid goal-directed motor tasks such as braking while driving a car or taking a corrective step to keep balance on a moving bus. Previous research on motor deficits post-stroke focused on a decline in motor accuracy averaged across multiple trials [46,53,54]. In contrast, our study investigated intra-individual variability in motor accuracy by capturing trial-to-trial fluctuations in executing a goal-directed task. The stroke group demonstrated a 15.6% increase in the IIV of the endpoint error compared to the control group, suggesting reduced consistency in achieving desired accuracy during goal-directed ankle movements relative to age-matched healthy controls. Consistent with prior studies, we also noted an increased mean endpoint error in the stroke group (9.2% more than the control group), indicating reduced average accuracy in executing goal-directed ankle contractions [46]. Previous research on intra-individual variability in motor tasks has predominantly focused on older adults, attributing age-related increases in trial-to-trial variability to altered muscle activation with aging [41,45]. Given that stroke directly affects neuroanatomical integrity and interferes with descending motor commands, it is reasonable to anticipate an impact on the consistency of motor performance. An individual with high intra-individual variability may perform a motor task accurately one moment but may experience difficulty the next moment. Such unpredictability in motor performance compromises the ability to adapt to varying environments and may predispose individuals to a greater risk of accidents. Importantly, although the precise brain areas involved in increased IIV remain unclear, prior evidence suggests that central neurological dysfunction, rather than peripheral disturbances, is the likely source of variability [55]. To our knowledge, the current study presents the first empirical evidence that stroke-related motor impairments extend to trial-to-trial consistency of executing goal-directed motor tasks with direct implications on functional capacity post-stroke, suggesting both a specific aspect of vulnerability and a possible target for intervention.

### 5.2. Stroke-Related Increase in IIV in Motor Performance Relates to Postural Balance

A notable finding of our study was that greater intra-individual variability in the endpoint error predicted poor posture control. To understand how an increased IIV of motor performance in stroke affects the ability to perform functional mobility tasks, we examined the relationship between the IIV of the endpoint error with postural control in a quiet stance. In the stroke group, an increased IIV of the endpoint error predicted 33% of the variance in the sway area during quiet standing. A larger than typical effect size R^2^ of 33% suggests that a stroke-related increase in IIV has a significant and substantial impact on postural control. Importantly, the mean endpoint error did not significantly account for any additional variance in postural sway in the stroke group. Thus, postural control was predicted by the trial-to-trial consistency in performing goal-directed ankle movements rather than the mean-level accuracy of the goal-directed motor task. Overall, reduced consistency in performing goal-directed movements had an adverse effect on the ability to maintain balance in a standing position in individuals with stroke.

During quiet standing, an individual’s body is constantly correcting its posture by utilizing proprioceptive feedback from ankle joints. Stroke-specific impairments in muscle activations patterns, including increased ankle plantar flexor spasticity, reduced muscle strength, and co-contraction of ankle muscles, have been related to increased postural sway after stroke [56,57,58]. Here, we quantified the consistency and average accuracy of ankle motor control and its impact on postural sway. It is important to note that the stroke cohort in our study had relatively high functional ability in balance and walking with low-to-moderate motor impairments (Mean FMA-LE = 26.39/34). Therefore, the stroke group showed a trend toward increased sway area that did not emerge to be statistically significant. Further, higher functional status may result in improved mean-level motor accuracy. Perhaps, with greater recovery in generating accurate movements, the variability of movement across several repetitions becomes a more important factor for postural control in stroke. This view is supported by previous studies in older adults that show poor balance control is related to increased IIV in voluntary stepping reaction time [59]. Further older individuals who are at risk for falls demonstrated increased IIV in choice reaction time compared to non-fallers [60]. Taken together, the current and previous findings point toward the idea that heightened IIV in motor tasks may be linked to greater vulnerability in postural control.

### 5.3. Stroke-Related Increase in IIV in Motor Performance Relates to Delayed Braking

Driving is an important aspect of functional mobility that is often impacted by motor impairments after stroke [61,62,63]. We examined whether the speed of braking in response to a sudden visual stimulus during driving is associated with IIV in motor performance after stroke. As expected, participants in the stroke group demonstrated delayed braking response time in a simulated driving environment compared to healthy controls. These findings align with previous studies that have shown impaired driving performance following stroke [62,64,65,66,67,68].

A compelling finding from our study is that the IIV of the endpoint error predicted 27% of the variance in braking response time in the stroke group, independent of the mean endpoint error. A large effect size of 27% for R^2^ indicates that IIV is a powerful predictor of braking performance. Braking requires the ability to perform precisely timed ankle movements to keep a safe distance from the vehicle in the front. Specifically in our study, participants performed 10 trials of the braking task that required repeated, accurate movements of the lower limb. Diminished consistency in accurately executing goal-directed ankle movements may increase the likelihood of an adverse driving event because of a delayed or imprecise braking action. A recent study supports this view by demonstrating that increased motor variability during isometric force control is associated with delayed reaction time in older adults [69]. Another study showed that increased IIV in processing time predicted poor outcomes in flying simulator performance among middle-aged and older pilots [70]. Perhaps, impaired neural drive increases the likelihood of inconsistent motor performance that potentially compromises the ability to respond to a sudden stimulus. Overall, our findings highlight that reduced consistency in performing repeated goal-directed ankle movement negatively impacts braking performance. These findings are in line with self-reports of reduced participation in road driving behavior by the stroke group as indicated by their lower DHQ scores. Reduced consistency in executing accurate ankle movements may lead to failures in applying the brakes at critical moments. Therefore, a single missed or incorrect braking performance can be consequential and result in a driving accident or even a crash.

### 5.4. Considerations and Clinical Implications

A critical question to consider is whether the clinical etiology of stroke alters IIV in motor performance. The severity of stroke as measured with FMA, time since stroke, and age did not relate with the IIV in motor performance in our study sample. However, one study limitation is that we did not examine the influence of other factors such as lesion location, size of the damaged brain area, and time since stroke on IIV in motor performance, a key area for future investigations. Another limitation is that our study included a relatively small sample of stroke participants, which may affect the results and the effect sizes. Therefore, these findings should be interpreted with caution. A relatively homogenous group of high-functioning individuals with strokes may lead to an underestimation of the predictive relevance of IIV, compared to a more heterogenous group that includes participants with lower functional ability. Even though high-functioning individuals have the overall ability to maintain balance and drive, the inconsistency in motor performance may explain why they may be more susceptible to unexpected falls and driving accidents. Therefore, one possibility is that despite apparent functional recovery, inconsistent motor performance potentially makes moderately impaired stroke survivors more susceptible to environmental and internal stressors. Future studies are needed to confirm this explanation and investigate the relationship between IIV in motor performance and functional mobility in more severely impaired stroke individuals. Another limitation is the absence of pre-stroke performance data, which could more conclusively demonstrate whether the findings of increased IIV can be truly attributed to the stroke. One proposed solution is to prospectively track individuals at high risk of stroke before the onset of a cerebrovascular accident to gain a clearer insight into the stroke’s impact on IIV and its implication on functional mobility.

Currently, stroke motor rehabilitation largely focuses on the simple goal of achieving higher average performance in certain motor tasks rather than improving the consistency of quality movements that lead to task success [71,72]. One way of improving performance on a specific task could be by lowering trial-to-trial variability on task-related dimensions and, thus, creating greater consistency. For example, training-related reduction in trial-to-trial variability in movement trajectory in healthy adults improved movement times of wrist flexion–extension [73]. Further, reduced execution variability following training was correlated with improved adaptation and learning rates, with these gains retained after six months in individuals with cerebral palsy [74]. Similarly, individuals with stroke improved reaching accuracy by reducing intra-individual variability at the trained movement speed [75]. Our results showing the association between IIV in motor performance and functional mobility point to the possibility that lowering trial-to-trial variability of limb movements may be a crucial element for achieving optimal functional performance after stroke.

## 6. Conclusions

Overall, our findings highlight that reduced consistency in performing repeated goal-directed ankle movements was associated with postural control and delayed braking response. Relative to the conventional methods of quantifying motor impairments after stroke by using mean performance measures, we showed that trial-to-trial variability in motor performance was amplified following stroke. The impact of stroke on the intra-individual variability of motor performance far exceeded the impact of stroke on mean motor performance. The large effect sizes suggest that increased intra-individual variability in endpoint accuracy has a significant and substantial impact on postural sway and delayed braking response time in simulated driving in stroke survivors. These findings confirm that reduced within-person consistency in motor performance across trials is closely linked with deteriorated functional mobility. Understanding the importance of trial-to-trial consistency in motor performance and its association with functional performance after stroke may facilitate the development of effective rehabilitation interventions that target improved movement consistency to enhance overall functional recovery after stroke.

## Figures and Tables

**Table 1:** Demographics of the stroke and control groups.

Participant Characteristics	Stroke (*n* = 20)	Control (*n* = 20)
Age (years)	62.01 ± 14.39	62.01 ± 10.18
Sex (Male/Female), *n*	12/8	10/10
Fugl–Meyer Assessment-lower extremity (/34)	26.39 ± 6.33	n/a
Montreal Cognitive Assessment (/30)	21.90 ± 4.33	26.65 ± 2.18
Driving Habits Questionnaire (/15)	8.25 ± 5.80	13.95 ± 4.34
No. of individuals using walking aid	2	0
Hemiparetic side (left/right), *n*	11/9	n/a
Time since stroke (years)	4.85 ± 3.95	n/a
Stroke location	12 cortical, 3 sub-cortical, 5 unavailable	n/a

**Table 2:** Raw scores of motor performance on goal-directed tasks, postural sway, and braking for the two groups.

	Stroke (*n* = 20)	Control (*n* = 20)
Endpoint error (%)		
Mean	83.66 ± 66.23	52.54 ± 30.58
Within-person SD	53.66 ± 37.29	30.16 ± 19.32
Sway area (cm^2^/s^5^)	40.97 ± 40.92	29.09 ± 19.59
Braking response time (ms)	910 ± 200	790 ± 110

**Table 3:** Summary of hierarchical regression for predicting sway area in the stroke group.

Model Summary							
Variable	*B*	95% CI for B		SE *B*	β	*R* ^2^	Δ*R*^2^
		LL	UL				
Step 1						0.327 **	0.327 **
Constant	0.559	−0.114	1.232	0.320			
IIV of endpoint error	0.571	0.165	0.977	0.193	0.572		
Step 2						0.017 *	0.017 *
Constant	0.719	−0.137	1.575	0.406			
IIV of endpoint error	0.701	0.114	1.288	0.278	0.702		
Mean endpoint error	−0.204	−0.855	0.448	0.309	−0.184		

Note. *n* = 20. We examined whether IIV of endpoint error and/or mean of endpoint error predicted the sway area. We entered the IIV in endpoint error (step 1) and mean endpoint error (step 2) to predict the sway area among the stroke group. CI = confidence interval; LL = lower limit; UL = upper limit. * *p* < 0.05. ** *p* < 0.01.

**Table 4:** Summary of hierarchical regression for predicting braking response time in the stroke group.

Model Summary							
Variable	*B*	95% CI for B		SE *B*	β	*R* ^2^	Δ*R*^2^
		LL	UL				
Step 1						0.265	0.265 *
Constant	2.725	2.531	2.919	0.092			
IIV of endpoint error	0.136	0.020	0.252	0.055	0.515		
Step 2						0.296	0.031
Constant	2.663	2.412	2.915	0.119			
IIV of endpoint error	0.093	−0.067	0.253	0.076	0.351		
Mean endpoint error	0.072	−0.110	0.254	0.086	0.240		

Note. *n* = 20. We examined whether the IIV of endpoint error and/or mean of endpoint error predicted the braking response time. We entered the IIV in endpoint error (step 1) and mean endpoint error (step 2) to predict the braking response time. CI = confidence interval; LL =lower limit; UL = upper limit. * *p* < 0.05.

## Data Availability

Data and materials can be provided upon request.

## Abbreviations

CI	confidence interval
cm	centimeters
DHQ	Driving Habits Questionnaire
FMA	Fugl–Meyer Assessment
IIV	intra-individual variability
LL	lower limit
MoCA	Montreal Cognitive Assessment
ms	milliseconds
s	seconds
SD	standard deviation
UL	upper limit

## References

[1] Patel A.T., Duncan P.W., Lai S.M., Studenski S. (2000). The relation between impairments and functional outcomes poststroke. Arch. Phys. Med. Rehab..

[2] Rafsten L., Meirelles C., Danielsson A., Sunnerhagen K.S. (2019). Impaired Motor Function in the Affected Arm Predicts Impaired Postural Balance After Stroke: A Cross Sectional Study. Front. Neurol..

[3] Eng J.J., Kim C.M., Macintyre D.L. (2002). Reliability of lower extremity strength measures in persons with chronic stroke. Arch. Phys. Med. Rehabil..

[4] Rohrer B., Fasoli S., Krebs H.I., Hughes R., Volpe B., Frontera W.R., Stein J., Hogan N. (2002). Movement smoothness changes during stroke recovery. J. Neurosci..

[5] Lam T.K., Binns M.A., Honjo K., Dawson D.R., Ross B., Stuss D.T., Black S.E., Chen J.J., Fujioka T., Chen J.L. (2018). Variability in stroke motor outcome is explained by structural and functional integrity of the motor system. Sci. Rep..

[6] Nesselroade J.R., Salthouse T.A. (2004). Methodological and theoretical implications of intraindividual variability in perceptual-motor performance. J. Gerontol. B Psychol. Sci. Soc. Sci..

[7] Yacoubi B., Christou E.A. (2024). Motor Output Variability in Movement Disorders: Insights from Essential Tremor. Exerc. Sport Sci. Rev..

[8] Munsell E.G.S., Bui Q., Kaufman K.J., Tomazin S.E., Regan B.A., Lenze E.J., Lee J.M., Mohr D.C., Fong M.W.M., Metts C.L. (2024). Intraindividual variability in post-stroke cognition and its relationship with activities of daily living and social functioning: An ecological momentary assessment approach. Top. Stroke Rehabil..

[9] Jackson J.D., Balota D.A., Duchek J.M., Head D. (2012). White matter integrity and reaction time intraindividual variability in healthy aging and early-stage Alzheimer disease. Neuropsychologia.

[10] MacDonald S.W., Nyberg L., Backman L. (2006). Intra-individual variability in behavior: Links to brain structure, neurotransmission and neuronal activity. Trends Neurosci..

[11] Fjell A.M., Westlye L.T., Amlien I.K., Walhovd K.B. (2011). Reduced White Matter Integrity Is Related to Cognitive Instability. J. Neurosci..

[12] Stuss D.T., Pogue J., Buckle L., Bondar J. (1994). Characterization of Stability of Performance in Patients with Traumatic Brain Injury: Variability and Consistency on Reaction Time Tests. Neuropsychology.

[13] Burton C.L., Hultsch D.F., Strauss E., Hunter M.A. (2002). Intraindividual variability in physical and emotional functioning: Comparison of adults with traumatic brain injuries and healthy adults. Clin. Neuropsychol..

[14] Burton C.L., Strauss E., Hultsch D.F., Moll A., Hunter M.A. (2006). Intraindividual variability as a marker of neurological dysfunction: A comparison of Alzheimer’s disease and Parkinson’s disease. J. Clin. Exp. Neuropsyc..

[15] Davis J.J., Sivaramakrishnan A., Rolin S., Subramanian S. (2023). Intra-individual variability in cognitive performance predicts functional decline in Parkinson’s disease. Appl. Neuropsych.-Adul..

[16] Wojtowicz M., Berrigan L.I., Fisk J.D. (2012). Intra-individual Variability as a Measure of Information Processing Difficulties in Multiple Sclerosis. Int. J. MS Care.

[17] Mazerolle E.L., Wojtowicz M.A., Omisade A., Fisk J.D. (2013). Intra-individual variability in information processing speed reflects white matter microstructure in multiple sclerosis. Neuroimage Clin..

[18] Costa A.S., Dogan I., Schulz J.B., Reetz K. (2019). Going beyond the mean: Intraindividual variability of cognitive performance in prodromal and early neurodegenerative disorders. Clin. Neuropsychol..

[19] Mumme R., Pushpanathan M., Donaldson S., Weinborn M., Rainey-Smith S.R., Maruff P., Bucks R.S. (2021). Longitudinal Association of Intraindividual Variability with Cognitive Decline and Dementia: A Meta-Analysis. Neuropsychology.

[20] Kawahira K., Shimodozono M., Ogata A., Etoh S., Ikeda S., Yoshida A., Tanaka N., Tsujio S. (2005). Impaired visuo-motor skills in the unaffected lower limb of patients with stroke. Int. J. Neurosci..

[21] Lindberg P.G., Roche N., Robertson J., Roby-Brami A., Bussel B., Maier M.A. (2012). Affected and unaffected quantitative aspects of grip force control in hemiparetic patients after stroke. Brain Res..

[22] Mukherjee M., Koutakis P., Siu K.C., Fayad P.B., Stergiou N. (2013). Stroke Survivors Control the Temporal Structure of Variability During Reaching in Dynamic Environments. Ann. Biomed. Eng..

[23] Sendhilnathan N., Basu D., Murthy A. (2020). Assessing within-trial and across-trial neural variability in macaque frontal eye fields and their relation to behaviour. Eur. J. Neurosci..

[24] Ranganathan R., Gebara R., Andary M., Sylvain J. (2019). Chronic stroke survivors show task-dependent modulation of motor variability during bimanual coordination. J. Neurophysiol..

[25] Lang C.E., Wagner J.M., Bastian A.J., Hu Q.L., Edwards D.F., Sahrmann S.A., Dromerick A.W. (2005). Deficits in grasp versus reach during acute hemiparesis. Exp. Brain Res..

[26] Roalf D.R., Quarmley M., Mechanic-Hamilton D., Wolk D.A., Arnold S.E., Moberg P.J. (2016). Alzheimer’s Disease Neuroimaging I: Within-Individual Variability: An Index for Subtle Change in Neurocognition in Mild Cognitive Impairment. J. Alzheimer’s Dis..

[27] Haynes B.I., Bauermeister S., Bunce D. (2017). A Systematic Review of Longitudinal Associations Between Reaction Time Intraindividual Variability and Age-Related Cognitive Decline or Impairment, Dementia, and Mortality. J. Int. Neuropsych. Soc..

[28] Castellanos F.X., Sonuga-Barke E.J.S., Scheres A., Di Martino A., Hyde C., Walters J.R. (2005). Varieties of attention-deficit/hyperactivity disorder-related intra-individual variability. Biol. Psychiat..

[29] MacPherson S.E., Gillebert C.R., Robinson G.A., Vallesi A. (2019). Editorial: Intra- and Inter-individual Variability of Executive Functions: Determinant and Modulating Factors in Healthy and Pathological Conditions. Front. Psychol..

[30] Borges S.D., Radanovic M., Forlenza O.V. (2018). Correlation between functional mobility and cognitive performance in older adults with cognitive impairment. Aging Neuropsychol. C.

[31] Gothe N.P., Fanning J., Awick E., Chung D., Wójcicki T.R., Olson E.A., Mullen S.P., Voss M., Erickson K.I., Kramer A.F. (2014). Executive Function Processes Predict Mobility Outcomes in Older Adults. J. Am. Geriatr. Soc..

[32] Webber S.C., Porter M.M., Menec V.H. (2010). Mobility in older adults: A comprehensive framework. Gerontologist.

[33] Garland S.J., Ivanova T.D., Mochizuki G. (2007). Recovery of standing balance and health-related quality of life after mild or moderately severe stroke. Arch. Phys. Med. Rehabil..

[34] Griffen J.A., Rapport L.J., Bryer R.C., Scott C.A. (2009). Driving status and community integration after stroke. Top. Stroke Rehabil..

[35] Bielak A.A., Hultsch D.F., Strauss E., Macdonald S.W., Hunter M.A. (2010). Intraindividual variability in reaction time predicts cognitive outcomes 5 years later. Neuropsychology.

[36] Lövdén M., Li S.C., Shing Y.L., Lindenberger U. (2007). Within-person trial-to-trial variability precedes and predicts cognitive decline in old and very old age: Longitudinal data from the Berlin Aging Study. Neuropsychologia.

[37] Gladstone D.J., Danells C.J., Black S.E. (2002). The Fugl-Meyer Assessment of motor recovery after stroke: A critical review of its measurement properties. Neurorehab. Neural Repair.

[38] Nasreddine Z.S., Phillips N.A., Bedirian V., Charbonneau S., Whitehead V., Collin I., Cummings J.L., Chertkow H. (2005). The Montreal Cognitive Assessment, MoCA: A brief screening tool for mild cognitive impairment. J. Am. Geriatr. Soc..

[39] Owsley C., Stalvey B., Wells J., Sloane M.E. (1999). Older drivers and cataract: Driving habits and crash risk. J. Gerontol. A Biol. Sci. Med. Sci..

[40] Casamento-Moran A., Chen Y.T., Kwon M., Snyder A., Subramony S.H., Vaillancourt D.E., Christou E.A. (2015). Force dysmetria in spinocerebellar ataxia 6 correlates with functional capacity. Front. Hum. Neurosci..

[41] Christou E.A., Poston B., Enoka J.A., Enoka R.M. (2007). Different neural adjustments improve endpoint accuracy with practice in young and old adults. J. Neurophysiol..

[42] Casamento-Moran A., Chen Y.T., Lodha N., Yacoubi B., Christou E.A. (2017). Motor plan differs for young and older adults during similar movements. J. Neurophysiol..

[43] Casamento-Moran A., Fleeman R., Chen Y.T., Kwon M., Fox E.J., Yacoubi B., Christou E.A. (2018). Neuromuscular variability and spatial accuracy in children and older adults. J. Electromyogr. Kinesiol..

[44] Casamento-Moran A., Hunter S.K., Chen Y.T., Kwon M.H., Fox E.J., Yacoubi B., Christou E.A. (2017). Sex differences in spatial accuracy relate to the neural activation of antagonistic muscles in young adults. Exp. Brain Res..

[45] Delmas S., Choi Y.J., Komer M., Weintraub M., Yacoubi B., Christou E.A. (2021). Older adults use a motor plan that is detrimental to endpoint control. Sci. Rep..

[46] Lodha N., Patel P., Casamento-Moran A., Gauger K., Christou E.A. (2019). Endpoint accuracy of goal-directed ankle movements correlates to over-ground walking in stroke. Clin. Neurophysiol..

[47] Jung H.G., Yamasaki M. (2016). Association of lower extremity range of motion and muscle strength with physical performance of community-dwelling older women. J. Physiol. Anthropol..

[48] Shumway-Cook A., Woollacott M.H. (2007). Motor Control: Translating Research into Clinical Practice.

[49] Mancini M., Horak F.B. (2016). Potential of APDM mobility lab for the monitoring of the progression of Parkinson’s disease. Expert Rev. Med. Devic..

[50] Morris R., Stuart S., McBarron G., Fino P.C., Mancini M., Curtze C. (2019). Validity of Mobility Lab (version 2) for gait assessment in young adults, older adults and Parkinson’s disease. Physiol. Meas..

[51] Cohen J. (1988). Statistical Power Analysis for the Behavioral Sciences.

[52] Altenburger P., Ambike S.S., Haddad J.M. (2023). Integrating Motor Variability Evaluation Into Movement System Assessment. Phys. Ther..

[53] Schaefer S.Y., Mutha P.K., Haaland K.Y., Sainburg R.L. (2012). Hemispheric specialization for movement control produces dissociable differences in online corrections after stroke. Cereb. Cortex.

[54] Winstein C.J., Pohl P.S. (1995). Effects of unilateral brain damage on the control of goal-directed hand movements. Exp. Brain Res..

[55] Hultsch D.F., MacDonald S.W., Hunter M.A., Levy-Bencheton J., Strauss E. (2000). Intraindividual variability in cognitive performance in older adults: Comparison of adults with mild dementia, adults with arthritis, and healthy adults. Neuropsychology.

[56] Marigold D.S., Eng J.J., Tokuno C.D., Donnelly C.A. (2004). Contribution of muscle strength and integration of afferent input to postural instability in persons with stroke. Neurorehabil. Neural Repair.

[57] Garland S.J., Gray V.L., Knorr S. (2009). Muscle activation patterns and postural control following stroke. Motor Control.

[58] Rahimzadeh Khiabani R., Mochizuki G., Ismail F., Boulias C., Phadke C.P., Gage W.H. (2017). Impact of Spasticity on Balance Control during Quiet Standing in Persons after Stroke. Stroke Res. Treat..

[59] Bunce D., Haynes B.I., Lord S.R., Gschwind Y.J., Kochan N.A., Reppermund S., Brodaty H., Sachdev P.S., Delbaere K. (2017). Intraindividual Stepping Reaction Time Variability Predicts Falls in Older Adults with Mild Cognitive Impairment. J. Gerontol. A Biol. Sci. Med. Sci..

[60] Reelick M.F., Kessels R.P., Faes M.C., Weerdesteyn V., Esselink R.A., Olde Rikkert M.G. (2011). Increased intra-individual variability in stride length and reaction time in recurrent older fallers. Aging Clin. Exp. Res..

[61] Aufman E.L., Bland M.D., Barco P.P., Carr D.B., Lang C.E. (2013). Predictors of return to driving after stroke. Am. J. Phys. Med. Rehabil..

[62] Lodha N., Patel P., Casamento-Moran A., Hays E., Poisson S.N., Christou E.A. (2018). Strength or Motor Control: What Matters in High-Functioning Stroke?. Front. Neurol..

[63] Patel P., Alam T., Tracy B.L., Lodha N. (2021). Impaired force control contributes to car steering dysfunction in chronic stroke. Disabil. Rehabil..

[64] Devos H., Tant M., Akinwuntan A.E. (2014). On-road driving impairments and associated cognitive deficits after stroke. Cerebrovasc. Dis..

[65] Hird M.A., Vetivelu A., Saposnik G., Schweizer T.A. (2014). Cognitive, on-road, and simulator-based driving assessment after stroke. J. Stroke Cerebrovasc. Dis..

[66] Lodha N., Patel P., Shad J.M., Casamento-Moran A., Christou E.A. (2021). Cognitive and motor deficits contribute to longer braking time in stroke. J. Neuroeng. Rehabil..

[67] Perrier M.J., Korner-Bitensky N., Mayo N.E. (2010). Patient factors associated with return to driving poststroke: Findings from a multicenter cohort study. Arch. Phys. Med. Rehabil..

[68] Hird M.A., Vesely K.A., Christie L.E., Alves M.A., Pongmoragot J., Saposnik G., Schweizer T.A. (2015). Is it safe to drive after acute mild stroke? A preliminary report. J. Neurol. Sci..

[69] Kwon M., Christou E.A. (2018). Visual information processing in older adults: Reaction time and motor unit pool modulation. J. Neurophysiol..

[70] Kennedy Q., Taylor J., Heraldez D., Noda A., Lazzeroni L.C., Yesavage J. (2013). Intraindividual variability in basic reaction time predicts middle-aged and older pilots’ flight simulator performance. J. Gerontol. B Psychol. Sci. Soc. Sci..

[71] Duncan P.W., Sullivan K.J., Behrman A.L., Azen S.P., Wu S.S., Nadeau S.E., Dobkin B.H., Rose D.K., Tilson J.K., Cen S. (2011). Body-weight-supported treadmill rehabilitation after stroke. N. Engl. J. Med..

[72] Hornby T.G., Henderson C.E., Plawecki A., Lucas E., Lotter J., Holthus M., Brazg G., Fahey M., Woodward J., Ardestani M. (2019). Contributions of Stepping Intensity and Variability to Mobility in Individuals Poststroke. Stroke.

[73] Shmuelof L., Krakauer J.W., Mazzoni P. (2012). How is a motor skill learned? Change and invariance at the levels of task success and trajectory control. J. Neurophysiol..

[74] Mawase F., Bar-Haim S., Joubran K., Rubin L., Karniel A., Shmuelof L. (2016). Increased Adaptation Rates and Reduction in Trial-by-Trial Variability in Subjects with Cerebral Palsy Following a Multi-session Locomotor Adaptation Training. Front. Hum. Neurosci..

[75] Hammerbeck U., Yousif N., Hoad D., Greenwood R., Diedrichsen J., Rothwell J.C. (2017). Chronic Stroke Survivors Improve Reaching Accuracy by Reducing Movement Variability at the Trained Movement Speed. Neurorehabil. Neural Repair.

